# Supplementary material for: Bidirectional microwave-optical transduction based on integration of high-overtone bulk acoustic resonators and photonic circuits
Source: Nat Commun. 2024 Jul 19;15:6096. doi: 10.1038/s41467-024-49467-8 (PMC11271592; doi:10.1038/s41467-024-49467-8)
Supplement: Supplementary file 1 — Supplementary Information [file 41467_2024_49467_MOESM1_ESM.pdf]

Bidirectional microwave-optical transduction based on integration of high-overtone bulk acoustic resonators and photonic circuits

Terence Blésin,[1,2] Wil Kao,[1,2] Anat Siddharth,[1,2] Rui N. Wang,[1,2] Alaina Attanasio,[3] Hao Tian,[3] Sunil A. Bhave,[3,*] and Tobias J. Kippenberg[1,2,†]

[1] *Institute of Physics, Swiss Federal Institute of Technology Lausanne (EPFL), CH-1015 Lausanne, Switzerland*
[2] *Center of Quantum Science and Engineering (EPFL), CH-1015 Lausanne, Switzerland*
[3] *OxideMEMS lab, Purdue University, West Lafayette, IN, USA*

Appendix A: Theory

1. Coupled mode theory for optical modes hybridization

Using a pair of optical resonances increases the intracavity photon number for a given input optical power, resulting in an enhanced optomechanical interaction. Such an optical doublet is implemented here by evanescently coupling two micro-rings, forming a photonic molecule. Consider two bare optical modes with resonance frequencies ω_l and ω_r that correspond to the left and right micro-rings, respectively. The two modes couple with a rate J, which yields the Hamiltonian [1]

$$\hat{\mathcal{H}} = \hbar\omega_\mathrm{l}\hat{a}_\mathrm{l}^\dagger\hat{a}_\mathrm{l} + \hbar\omega_\mathrm{r}\hat{a}_\mathrm{r}^\dagger\hat{a}_\mathrm{r} - \hbar J\left(\hat{a}_\mathrm{l}\hat{a}_\mathrm{r}^\dagger + \hat{a}_\mathrm{l}^\dagger\hat{a}_\mathrm{r}\right) \tag{A1}$$

and the equations of motion

$$\begin{aligned}\frac{d}{dt}\hat{a}_\mathrm{l}(t) &= \left(-i\omega_\mathrm{l} - \frac{\kappa_\mathrm{l}}{2}\right)\hat{a}_\mathrm{l}(t) + iJ\hat{a}_\mathrm{r}(t),\\ \frac{d}{dt}\hat{a}_\mathrm{r}(t) &= \left(-i\omega_\mathrm{r} - \frac{\kappa_\mathrm{r}}{2}\right)\hat{a}_\mathrm{r}(t) + iJ\hat{a}_\mathrm{l}(t).\end{aligned} \tag{A2}$$

With Laplace transform, they can be rewritten in frequency domain as

$$\begin{pmatrix}\chi_\mathrm{l}^{-1}[s] & -iJ\\ -iJ & \chi_\mathrm{r}^{-1}[s]\end{pmatrix}\begin{pmatrix}\hat{a}_\mathrm{l}[s]\\ \hat{a}_\mathrm{r}[s]\end{pmatrix} = \begin{pmatrix}\hat{a}_\mathrm{l}(0)\\ \hat{a}_\mathrm{r}(0)\end{pmatrix}, \tag{A3}$$

where $\chi_o[s] = (s + i\omega_o + \kappa_o/2)^{-1}$, $o = \mathrm{l}, \mathrm{r}$ denote the susceptibilities. Diagonalizing the matrix yields the eigenvalues

$$\begin{aligned}\lambda_\pm &= \frac{\chi_\mathrm{l}^{-1} + \chi_\mathrm{r}^{-1}}{2} \pm \frac{1}{2}\sqrt{\left(\chi_\mathrm{l}^{-1} - \chi_\mathrm{r}^{-1}\right)^2 - 4J^2}\\ &= (s + i\bar{\omega} + \bar{\kappa}/2) \pm \frac{1}{2}\sqrt{\frac{\mu^2}{4} - \delta^2 + i\mu\delta - 4J^2}.\end{aligned} \tag{A4}$$

Here we define the average optical frequency $\bar{\omega} = (\omega_\mathrm{l} + \omega_\mathrm{r})/2$, average linewidth $\bar{\kappa} = (\kappa_\mathrm{l} + \kappa_\mathrm{r})/2$, relative detuning of the micro-rings $\delta = \omega_\mathrm{l} - \omega_\mathrm{r}$, and linewidth difference $\mu = \kappa_\mathrm{l} - \kappa_\mathrm{r}$. The difference in resonance frequency and linewidth of the two supermodes can be computed as

$$\Delta\omega = \frac{1}{\sqrt{2}}\sqrt{-\frac{\mu^2}{4} + \delta^2 + 4J^2 + \sqrt{\left[\left(2J - \frac{\mu}{2}\right)^2 + \delta^2\right]\left[\left(2J + \frac{\mu}{2}\right)^2 + \delta^2\right]}}, \tag{A5}$$

$$\Delta\kappa = \sqrt{2}\sqrt{\frac{\mu^2}{4} - \delta^2 - 4J^2 + \sqrt{\left[\left(2J - \frac{\mu}{2}\right)^2 + \delta^2\right]\left[\left(2J + \frac{\mu}{2}\right)^2 + \delta^2\right]}}, \tag{A6}$$

* bhave@purdue.edu
† tobias.kippenberg@epfl.ch

where $\lambda_+ - \lambda_- = \Delta\kappa/2 + i\Delta\omega$. In the strong coupling regime where $2J \gg \mu/2$, we simply have $\Delta\omega \approx \sqrt{4J^2 + \delta^2}$ and $\Delta\kappa \approx \mu\sqrt{1 - 4J^2/(4J^2 + \delta^2)}$. The two supermodes are thus centered at $\bar{\omega}$ with a splitting of $\Delta\omega$, resulting in susceptibilites

$$\chi_\pm[s] = \left[s + i\left(\bar{\omega} \pm \frac{\Delta\omega}{2}\right) + \frac{1}{2}\left(\bar{\kappa} \pm \frac{\Delta\kappa}{2}\right)\right]^{-1}. \tag{A7}$$

Solving for the eigenvectors gives the participation ratios of the bare cavity modes in the supermodes

$$\begin{aligned} u_\pm &= \frac{2J}{\sqrt{4J^2 + \left|\mu \mp \Delta\kappa/2 + i\left(\delta \mp \Delta\omega\right)\right|^2}} \exp\left(i\phi_\pm\right), \\ v_\pm &= \frac{\left|\mu \mp \Delta\kappa/2 + i\left(\delta \mp \Delta\omega\right)\right|}{\sqrt{4J^2 + \left|\mu \mp \Delta\kappa/2 + i\left(\delta \mp \Delta\omega\right)\right|^2}} \exp\left[i\phi_\pm + i \arg\frac{\mu \mp \Delta\kappa/2 + i\left(\delta \mp \Delta\omega\right)}{2iJ}\right], \end{aligned} \tag{A8}$$

where $|u_\pm|^2 + |v_\pm|^2 = 1$ and $\phi_\pm$ is a global phase factor. In particular, the supermode $\hat{a}_- = u_-\hat{a}_\mathrm{l} + v_-\hat{a}_\mathrm{r}$ has a resonance frequency $\omega_- = \bar{\omega} - \Delta\omega/2$ and linewidth $\kappa_- = \bar{\kappa} - \Delta\kappa/2$. It is referred to as the symmetric supermode. The rationale behind the nomenclature can be seen by considering the limit of zero detuning $\delta = 0$, where we simply have $\hat{a}_- = \left(\hat{a}_\mathrm{l} + \hat{a}_\mathrm{r}\right)/\sqrt{2}$ with $\omega_- = \bar{\omega} - J$ and $\kappa_- = \bar{\kappa}$. On the other hand, the antisymmetric supermode $\hat{a}_+ = u_+\hat{a}_\mathrm{l} + v_+\hat{a}_\mathrm{r}$ has a resonance frequency $\omega_+ = \bar{\omega} + \Delta\omega/2$ and linewidth $\kappa_+ = \bar{\kappa} + \Delta\kappa/2$. When $\delta = 0$, this reduces to $\hat{a}_+ = \left(\hat{a}_\mathrm{l} - \hat{a}_\mathrm{r}\right)/\sqrt{2}$ with $\omega_+ = \bar{\omega} + J$ and $\kappa_+ = \bar{\kappa}$.

2. Transducer Hamiltonian and input-output relations

In terms of bare micro-ring modes, the transducer Hamiltonian is given by

$$\hat{\mathcal{H}} = \hbar\omega_\mathrm{m}\hat{b}^\dagger\hat{b} + \hbar\omega_\mathrm{l}\hat{a}_\mathrm{l}^\dagger\hat{a}_\mathrm{l} + \hbar\omega_\mathrm{r}\hat{a}_\mathrm{r}^\dagger\hat{a}_\mathrm{r} - \hbar J\left(\hat{a}_\mathrm{l}^\dagger\hat{a}_\mathrm{r} + \hat{a}_\mathrm{r}^\dagger\hat{a}_\mathrm{l}\right) - \hbar g_0\hat{a}_\mathrm{l}^\dagger\hat{a}_\mathrm{l}\left(\hat{b} + \hat{b}^\dagger\right) + \hat{\mathcal{H}}_\mathrm{drive}. \tag{A9}$$

Note that the piezo-coupled acoustic mode interacts with only one of the micro-rings. To compute quantities directly related to the experiments, we rewrite the Hamiltonian in terms of the supermodes (Appendix A 1) as

$$\hat{\mathcal{H}} = \hbar\omega_\mathrm{m}\hat{b}^\dagger\hat{b} + \hbar\omega_-\hat{a}_-^\dagger\hat{a}_- + \hbar\omega_+\hat{a}_+^\dagger\hat{a}_+ - \hbar g_0\left(|x|^2\hat{a}_-^\dagger\hat{a}_- + |y|^2\hat{a}_+^\dagger\hat{a}_+ + xy^*\hat{a}_+^\dagger\hat{a}_- + x^*y\hat{a}_-^\dagger\hat{a}_+\right)\left(\hat{b} + \hat{b}^\dagger\right) + \hat{\mathcal{H}}_\mathrm{drive} \tag{A10}$$

such that $\hat{a}_\mathrm{l} = x\hat{a}_- + y\hat{a}_+$. The driving term is given by

$$\begin{aligned} \hat{\mathcal{H}}_\mathrm{drive} = {} & i\hbar\sqrt{\kappa_\mathrm{ex,-}}\left(\hat{a}_\mathrm{in}\hat{a}_-^\dagger e^{-i\omega_\mathrm{L}t} - \hat{a}_\mathrm{in}^\dagger\hat{a}_- e^{i\omega_\mathrm{L}t}\right) + i\hbar\sqrt{\kappa_\mathrm{ex,+}}\left(\hat{a}_\mathrm{in}\hat{a}_+^\dagger e^{-i\omega_\mathrm{L}t} - \hat{a}_\mathrm{in}^\dagger\hat{a}_+ e^{i\omega_\mathrm{L}t}\right) \\ & + i\hbar\sqrt{\kappa_\mathrm{ex,m}}\left(\hat{c}_\mathrm{in}\hat{b}^\dagger e^{i\omega_\mathrm{m}t} - \hat{c}_\mathrm{in}^\dagger\hat{b}e^{-i\omega_\mathrm{m}t}\right) \end{aligned} \tag{A11}$$

for input optical and microwave fields $\hat{a}_\mathrm{in}$ and $\hat{c}_\mathrm{in}$. By going into the frames rotating at the laser drive frequency ω_L, we obtain

$$\begin{aligned} \hat{\mathcal{H}} = {} & \hbar\omega_\mathrm{m}\hat{b}^\dagger\hat{b} - \hbar\Delta_-\hat{a}_-^\dagger\hat{a}_- - \hbar\Delta_+\hat{a}_+^\dagger\hat{a}_+ \\ & - \hbar g_0\left(|x|^2\hat{a}_-^\dagger\hat{a}_- + |y|^2\hat{a}_+^\dagger\hat{a}_+\right)\left(\hat{b} + \hat{b}^\dagger\right) - \hbar g_0\left(xy^*\hat{a}_+^\dagger\hat{a}_- + x^*y\hat{a}_-^\dagger\hat{a}_+\right)\left(\hat{b} + \hat{b}^\dagger\right) \\ & + i\hbar\sqrt{\kappa_\mathrm{ex,-}}\left(\hat{a}_\mathrm{in}\hat{a}_-^\dagger - \hat{a}_\mathrm{in}^\dagger\hat{a}_-\right) + i\hbar\sqrt{\kappa_\mathrm{ex,+}}\left(\hat{a}_\mathrm{in}\hat{a}_+^\dagger - \hat{a}_\mathrm{in}^\dagger\hat{a}_+\right) + i\hbar\sqrt{\kappa_\mathrm{ex,m}}\left(\hat{c}_\mathrm{in}\hat{b}^\dagger e^{i\omega_\mathrm{m}t} - \hat{c}_\mathrm{in}^\dagger\hat{b}e^{-i\omega_\mathrm{m}t}\right), \end{aligned} \tag{A12}$$

where $\Delta_\pm = \omega_\mathrm{L} - \omega_\pm$. Considering small deviations of the supermodes around their steady-state amplitudes $\alpha_\pm \approx \left[\sqrt{\kappa_{\mathrm{ex},\pm}}/(\kappa_\pm/2 - i\Delta_\pm)\right] \sqrt{\dot{n}_\mathrm{in}^\pm} e^{i\phi_\pm}$ and letting the input field operators denote just the Langevin force, the Hamiltonian

$$\begin{aligned}\hat{\mathcal{H}} = {} & \hbar\omega_\mathrm{m}\hat{b}^\dagger\hat{b} - \hbar\Delta_-\hat{a}_-^\dagger\hat{a}_- - \hbar\Delta_+\hat{a}_+^\dagger\hat{a}_+ \\ & - \hbar g_0|x|^2\left(|\alpha_-|^2 + \alpha_-^*\hat{a}_- + \hat{a}_-^\dagger\alpha_- + \hat{a}_-^\dagger\hat{a}_-\right)\left(\hat{b} + \hat{b}^\dagger\right) \\ & - \hbar g_0|y|^2\left(|\alpha_+|^2 + \alpha_+^*\hat{a}_+ + \hat{a}_+^\dagger\alpha_+ + \hat{a}_+^\dagger\hat{a}_+\right)\left(\hat{b} + \hat{b}^\dagger\right) \\ & - \hbar g_0 xy^*\left(\alpha_+^*\alpha_- + \alpha_+^*\hat{a}_- + \hat{a}_+^\dagger\alpha_- + \hat{a}_+^\dagger\hat{a}_-\right)\left(\hat{b} + \hat{b}^\dagger\right) \\ & - \hbar g_0 x^* y\left(\alpha_-^*\alpha_+ + \alpha_-^*\hat{a}_+ + \hat{a}_-^\dagger\alpha_+ + \hat{a}_-^\dagger\hat{a}_+\right)\left(\hat{b} + \hat{b}^\dagger\right) \\ & + i\hbar\sqrt{\kappa_{\mathrm{ex},-}}\left(\hat{a}_\mathrm{in}\hat{a}_-^\dagger - \hat{a}_\mathrm{in}^\dagger\hat{a}_-\right) + i\hbar\sqrt{\kappa_{\mathrm{ex},+}}\left(\hat{a}_\mathrm{in}\hat{a}_+^\dagger - \hat{a}_\mathrm{in}^\dagger\hat{a}_+\right) \\ & + i\hbar\sqrt{\kappa_{\mathrm{ex,m}}}\left(\hat{c}_\mathrm{in}\hat{b}^\dagger e^{i\omega_\mathrm{m}t} - \hat{c}_\mathrm{in}^\dagger\hat{b}e^{-i\omega_\mathrm{m}t}\right)\end{aligned} \tag{A13}$$

can be further simplified. In the large intracavity photon number limit, the $\mathcal{O}\left(\hat{a}^2\right)$ terms are ommited as their contribution to the system dynamics is hidden by the $\mathcal{O}\left(\hat{a}\right)$ terms. The $\mathcal{O}\left(\alpha^2\right)$ constant terms are removed by shifting the position origin by the steady-state displacement

$$x_\mathrm{ss} = \frac{2g_0}{\omega_\mathrm{m}}\left(|x|^2|\alpha_-|^2 + |y|^2|\alpha_+|^2 + xy^*\alpha_+^*\alpha_- + x^*y\alpha_-^*\alpha_+\right)x_\mathrm{ZPF} \tag{A14}$$

using the translation operator $\hat{T}_{\hat{x}}(x_\mathrm{ss}) = \exp\left(x_\mathrm{ss}\hat{p}/(i\hbar)\right)$, $\hat{p}$ being the momentum operator associated with $\hat{x} = x_\mathrm{ZPF}\left(\hat{b} + \hat{b}^\dagger\right)$. The resulting effective Hamiltonian is then given by

$$\begin{aligned}\hat{\mathcal{H}} = {} & \hbar\omega_\mathrm{m}\hat{b}^\dagger\hat{b} - \hbar\Delta_-\hat{a}_-^\dagger\hat{a}_- - \hbar\Delta_+\hat{a}_+^\dagger\hat{a}_+ \\ & - \hbar g_0\left(|x|^2\alpha_-^* + xy^*\alpha_+^*\right)\hat{a}_-\left(\hat{b} + \hat{b}^\dagger\right) - \hbar g_0\left(|x|^2\alpha_- + x^*y\alpha_+\right)\hat{a}_-^\dagger\left(\hat{b} + \hat{b}^\dagger\right) \\ & - \hbar g_0\left(|y|^2\alpha_+^* + x^*y\alpha_-^*\right)\hat{a}_+\left(\hat{b} + \hat{b}^\dagger\right) - \hbar g_0\left(|y|^2\alpha_+ + xy^*\alpha_-\right)\hat{a}_+^\dagger\left(\hat{b} + \hat{b}^\dagger\right) \\ & + i\hbar\sqrt{\kappa_{\mathrm{ex},-}}\left(\hat{a}_\mathrm{in}\hat{a}_-^\dagger - \hat{a}_\mathrm{in}^\dagger\hat{a}_-\right) + i\hbar\sqrt{\kappa_{\mathrm{ex},+}}\left(\hat{a}_\mathrm{in}\hat{a}_+^\dagger - \hat{a}_\mathrm{in}^\dagger\hat{a}_+\right) + i\hbar\sqrt{\kappa_{\mathrm{ex,m}}}\left(\hat{c}_\mathrm{in}\hat{b}^\dagger e^{i\omega_\mathrm{m}t} - \hat{c}_\mathrm{in}^\dagger\hat{b}e^{-i\omega_\mathrm{m}t}\right).\end{aligned} \tag{A15}$$

Finally, by defining effective optomechanical coupling rates

$$\begin{aligned}g_- &= g_0\left(|x|^2\alpha_- + x^*y\alpha_+\right), \\ g_+ &= g_0\left(|y|^2\alpha_+ + xy^*\alpha_-\right),\end{aligned} \tag{A16}$$

and going into a rotating frame defined by the operator

$$\hat{U} = \exp\left(\frac{1}{i\hbar}\hbar\Delta_-\hat{a}_-^\dagger\hat{a}_- t\right)\exp\left(\frac{1}{i\hbar}\hbar\Delta_+\hat{a}_+^\dagger\hat{a}_+ t\right)\exp\left(\frac{-1}{i\hbar}\hbar\omega_\mathrm{m}\hat{b}^\dagger\hat{b}t\right), \tag{A17}$$

we have

$$\begin{aligned}\hat{\mathcal{H}} = {} & -\hbar\left(g_-^*\hat{a}_- e^{i\Delta_- t} + g_-\hat{a}_-^\dagger e^{-i\Delta_- t}\right)\left(\hat{b}e^{-i\omega_\mathrm{m}t} + \hat{b}^\dagger e^{i\omega_\mathrm{m}t}\right) \\ & - \hbar\left(g_+^*\hat{a}_+ e^{i\Delta_+ t} + g_+\hat{a}_+^\dagger e^{-i\Delta_+ t}\right)\left(\hat{b}e^{-i\omega_\mathrm{m}t} + \hat{b}^\dagger e^{i\omega_\mathrm{m}t}\right) \\ & + i\hbar\sqrt{\kappa_{\mathrm{ex},-}}\left(\hat{a}_\mathrm{in}\hat{a}_-^\dagger e^{-i\Delta_- t} - \hat{a}_\mathrm{in}^\dagger\hat{a}_- e^{i\Delta_- t}\right) + i\hbar\sqrt{\kappa_{\mathrm{ex},+}}\left(\hat{a}_\mathrm{in}\hat{a}_+^\dagger e^{-i\Delta_+ t} - \hat{a}_\mathrm{in}^\dagger\hat{a}_+ e^{i\Delta_+ t}\right) \\ & + i\hbar\sqrt{\kappa_{\mathrm{ex,m}}}\left(\hat{c}_\mathrm{in}\hat{b}^\dagger - \hat{c}_\mathrm{in}^\dagger\hat{b}\right)\end{aligned} \tag{A18}$$

in the interaction picture. The time evolution of the bosonic modes can be computed using the Heisenberg-Langevin equations given by

$$\begin{aligned}
\frac{d}{dt}\hat{a}_-(t) =&\frac{-\kappa_-}{2}\hat{a}_-(t) + ig_-\left[\hat{b}(t)e^{-i(\omega_\mathrm{m}+\Delta_-)t} + \hat{b}^\dagger(t)e^{i(\omega_\mathrm{m}-\Delta_-)t}\right] + \sqrt{\kappa_{\mathrm{ex},-}}\hat{a}_\mathrm{in}(t)e^{-i\Delta_- t},\\
\frac{d}{dt}\hat{a}_+(t) =&\frac{-\kappa_+}{2}\hat{a}_+(t) + ig_+\left[\hat{b}(t)e^{-i(\omega_\mathrm{m}+\Delta_+)t} + \hat{b}^\dagger(t)e^{i(\omega_\mathrm{m}-\Delta_+)t}\right] + \sqrt{\kappa_{\mathrm{ex},+}}\hat{a}_\mathrm{in}(t)e^{-i\Delta_+ t},\\
\frac{d}{dt}\hat{b}(t) =&\frac{-\kappa_\mathrm{m}}{2}\hat{b}(t) + ig_-^*\hat{a}_-(t)e^{i(\omega_\mathrm{m}+\Delta_-)t} + ig_-\hat{a}_-^\dagger(t)e^{i(\omega_\mathrm{m}-\Delta_-)t}\\
&+ ig_+^*\hat{a}_+(t)e^{i(\omega_\mathrm{m}+\Delta_+)t} + ig_+\hat{a}_+^\dagger(t)e^{i(\omega_\mathrm{m}-\Delta_+)t} + \sqrt{\kappa_{\mathrm{ex,m}}}\hat{c}_\mathrm{in}(t).
\end{aligned} \tag{A19}$$

We can move to the frequency domain by using the Fourier transform

$$\hat{a}[\omega] = \mathcal{F}\left(\hat{a}(t)\right)[\omega] = \int_{-\infty}^{\infty}\hat{a}(t)e^{i\omega t}dt \tag{A20}$$

along with the properties

$$\begin{aligned}
\mathcal{F}\left(\frac{d}{dt}\hat{a}(t)\right)[\omega] = -i\omega\hat{a}[\omega],\\
\mathcal{F}\left(\hat{a}(t)e^{-i\Delta t}\right)[\omega] = \hat{a}[\omega-\Delta],\\
\hat{a}^\dagger[\omega] \equiv \mathcal{F}\left(\hat{a}^\dagger(t)\right)[\omega] = \left(\hat{a}[-\omega]\right)^\dagger.
\end{aligned} \tag{A21}$$

We then have

$$\begin{aligned}
\hat{a}_-[\omega] &= ig_-\chi_-[\omega]\left(\hat{b}[\omega-(\omega_\mathrm{m}+\Delta_-)] + \hat{b}^\dagger[\omega+(\omega_\mathrm{m}-\Delta_-)]\right) + \sqrt{\kappa_{\mathrm{ex},-}}\chi_-[\omega]\hat{a}_\mathrm{in}[\omega-\Delta_-],\\
\hat{a}_+[\omega] &= ig_+\chi_+[\omega]\left(\hat{b}[\omega-(\omega_\mathrm{m}+\Delta_+)] + \hat{b}^\dagger[\omega+(\omega_\mathrm{m}-\Delta_+)]\right) + \sqrt{\kappa_{\mathrm{ex},+}}\chi_+[\omega]\hat{a}_\mathrm{in}[\omega-\Delta_+],\\
\hat{b}[\omega] &= ig_-^*\chi_\mathrm{m}[\omega]\hat{a}_-[\omega+(\omega_\mathrm{m}+\Delta_-)] + ig_-\chi_\mathrm{m}[\omega]\hat{a}_-^\dagger[\omega+(\omega_\mathrm{m}-\Delta_-)]\\
&\quad + ig_+^*\chi_\mathrm{m}[\omega]\hat{a}_+[\omega+(\omega_\mathrm{m}+\Delta_+)] + ig_+\chi_\mathrm{m}[\omega]\hat{a}_+^\dagger[\omega+(\omega_\mathrm{m}-\Delta_+)] + \sqrt{\kappa_{\mathrm{ex,m}}}\chi_\mathrm{m}[\omega]\hat{c}_\mathrm{in}[\omega],
\end{aligned} \tag{A22}$$

with the susceptibilities $\chi_-[\omega] = \left(-i\omega+\kappa_-/2\right)^{-1}$, $\chi_+[\omega] = \left(-i\omega+\kappa_+/2\right)^{-1}$ and $\chi_\mathrm{m}[\omega] = \left(-i\omega+\kappa_\mathrm{m}/2\right)^{-1}$. The bath operators are linked through the input-output relations

$$\begin{aligned}
\hat{a}_\mathrm{out}(t)e^{-i\omega_\mathrm{L}t} &= \hat{a}_\mathrm{in}(t)e^{-i\omega_\mathrm{L}t} - \sqrt{\kappa_{\mathrm{ex},-}}\hat{a}_-(t)e^{-i\omega_- t} - \sqrt{\kappa_{\mathrm{ex},+}}\hat{a}_+(t)e^{-i\omega_+ t},\\
\hat{c}_\mathrm{out}(t)e^{-i\omega_\mathrm{m}t} &= -\hat{c}_\mathrm{in}(t)e^{-i\omega_\mathrm{m}t} + \sqrt{\kappa_{\mathrm{ex,m}}}\hat{b}(t)e^{-i\omega_\mathrm{m}t}
\end{aligned} \tag{A23}$$

or

$$\begin{aligned}
\hat{a}_\mathrm{out}[\omega] &= \hat{a}_\mathrm{in}[\omega] - \sqrt{\kappa_{\mathrm{ex},-}}\hat{a}_-[\omega+\Delta_-] - \sqrt{\kappa_{\mathrm{ex},+}}\hat{a}_+[\omega+\Delta_+],\\
\hat{c}_\mathrm{out}[\omega] &= -\hat{c}_\mathrm{in}[\omega] + \sqrt{\kappa_{\mathrm{ex,m}}}\hat{b}[\omega]
\end{aligned} \tag{A24}$$

in frequency domain. They satisfy the commutation relations $\left[\hat{a}_\mathrm{in}[\omega], \hat{a}_\mathrm{in}^\dagger[\omega']\right] = \delta[\omega-\omega']$ and $\left[\hat{c}_\mathrm{in}[\omega], \hat{c}_\mathrm{in}^\dagger[\omega']\right] = \delta[\omega-\omega']$.

3. Conversion efficiency and added noise

a. *Anti-Stokes configuration*

For the following analysis, we consider the triply resonant configuration where $\omega_+ - \omega_- = \omega_\mathrm{m}$ and the optical pump is on resonance with one of the optical supermodes. First, we consider the anti-Stokes configuration where $\Delta_- = 0$ and $\Delta_+ = -\omega_\mathrm{m}$. From Eq. A24, we have the input-output relation

$$\hat{a}_\mathrm{out}[\omega] = \hat{a}_\mathrm{in}[\omega] - \sqrt{\kappa_{\mathrm{ex},-}}\hat{a}_-[\omega] - \sqrt{\kappa_{\mathrm{ex},+}}\hat{a}_+[\omega-\omega_\mathrm{m}]. \tag{A25}$$

The equations of motion is given by

$$\begin{aligned}
\hat{a}_-[\omega] =& \; ig_-\chi_-[\omega]\left(\hat{b}[\omega-\omega_\mathrm{m}]+\hat{b}^\dagger[\omega+\omega_\mathrm{m}]\right)+\sqrt{\kappa_{\mathrm{ex},-}}\chi_-[\omega]\hat{a}_\mathrm{in}[\omega] \\
\approx& \; \sqrt{\kappa_{\mathrm{ex},-}}\chi_-[\omega]\hat{a}_\mathrm{in}[\omega], \\
\hat{a}_+[\omega] =& \; ig_+\chi_+[\omega]\left(\hat{b}[\omega]+\hat{b}^\dagger[\omega+2\omega_\mathrm{m}]\right)+\sqrt{\kappa_{\mathrm{ex},+}}\chi_+[\omega]\hat{a}_\mathrm{in}[\omega+\omega_\mathrm{m}] \\
\approx& \; ig_+\chi_+[\omega]\hat{b}[\omega]+\sqrt{\kappa_{\mathrm{ex},+}}\chi_+[\omega]\hat{a}_\mathrm{in}[\omega+\omega_\mathrm{m}], \\
\hat{b}[\omega] =& \; ig_-^*\chi_\mathrm{m}[\omega]\hat{a}_-[\omega+\omega_\mathrm{m}]+ig_-\chi_\mathrm{m}[\omega]\hat{a}_-^\dagger[\omega+\omega_\mathrm{m}]+ig_+^*\chi_\mathrm{m}[\omega]\hat{a}_+[\omega]+ig_+\chi_\mathrm{m}[\omega]\hat{a}_+^\dagger[\omega+2\omega_\mathrm{m}]+\sqrt{\kappa_{\mathrm{ex,m}}}\chi_\mathrm{m}[\omega]\hat{c}_\mathrm{in}[\omega] \\
\approx& \; ig_+^*\chi_\mathrm{m}[\omega]\hat{a}_+[\omega]+\sqrt{\kappa_{\mathrm{ex,m}}}\chi_\mathrm{m}[\omega]\hat{c}_\mathrm{in}[\omega].
\end{aligned} \tag{A26}$$

Here we drop the terms that contain susceptibilities and ladder operators with an offsetted frequency dependence (with the exception of the bath operators), since they necessarily imply an attenuation of at least $\kappa_+/(2\omega_\mathrm{m})$ compared to the dominant terms. This approximation is valid in the resolved-sideband regime $\kappa_-,\kappa_+\ll\omega_\mathrm{m}$. Supplementary Figure 1a shows the signal flow graph representing the equations of motion, from which we deduced the transfer functions between the microwave field at ω_m and the optical field at $\omega_\mathrm{L}+\omega_\mathrm{m}$ using Mason's rule [2]

$$\begin{aligned}
S_{\hat{a}_\mathrm{out}[\omega+\omega_\mathrm{m}]\leftarrow\hat{c}_\mathrm{in}[\omega]} =& S_{\hat{c}_\mathrm{out}[\omega]\leftarrow\hat{a}_\mathrm{in}[\omega+\omega_\mathrm{m}]} = S^*_{\hat{a}^\dagger_\mathrm{out}[\omega+\omega_\mathrm{m}]\leftarrow\hat{c}^\dagger_\mathrm{in}[\omega]} = S^*_{\hat{c}^\dagger_\mathrm{out}[\omega]\leftarrow\hat{a}^\dagger_\mathrm{in}[\omega+\omega_\mathrm{m}]} \\
=& \frac{-i\sqrt{\kappa_{\mathrm{ex},+}}\sqrt{\kappa_{\mathrm{ex,m}}}g_+\chi_+[\omega]\chi_\mathrm{m}[\omega]}{1+|g_+|^2\chi_+[\omega]\chi_\mathrm{m}[\omega]}.
\end{aligned} \tag{A27}$$

The optical-optical and microwave-microwave transfer functions can also be derived as

$$S_{\hat{a}_\mathrm{out}[\omega+\omega_\mathrm{m}]\leftarrow\hat{a}_\mathrm{in}[\omega+\omega_\mathrm{m}]} = 1-\frac{\kappa_{\mathrm{ex},+}\chi_+[\omega]}{1+|g_+|^2\chi_+[\omega]\chi_\mathrm{m}[\omega]}-\kappa_{\mathrm{ex},-}\chi_-[\omega+\omega_\mathrm{m}], \tag{A28}$$

$$S_{\hat{c}_\mathrm{out}[\omega]\leftarrow\hat{c}_\mathrm{in}[\omega]} = -1+\frac{\kappa_{\mathrm{ex,m}}\chi_\mathrm{m}[\omega]}{1+|g_+|^2\chi_+[\omega]\chi_\mathrm{m}[\omega]}. \tag{A29}$$

The other transfer functions (e.g., $S_{\hat{a}^\dagger_\mathrm{out}[\omega+\omega_\mathrm{m}]\leftarrow\hat{c}_\mathrm{in}[\omega]}$) are expected to be small given the minuscule weight of the corresponding edges in the signal flow graph. The photon number flux at the optical output of the transducer is given by

$$\begin{aligned}
\hat{a}^\dagger_\mathrm{out}[\omega+\omega_\mathrm{m}]\hat{a}_\mathrm{out}[\omega+\omega_\mathrm{m}] \approx& \left(S^*_{\hat{a}_\mathrm{out}[\omega+\omega_\mathrm{m}]\leftarrow\hat{a}_\mathrm{in}[\omega+\omega_\mathrm{m}]}\hat{a}^\dagger_\mathrm{in}[\omega+\omega_\mathrm{m}]+S^*_{\hat{a}_\mathrm{out}[\omega+\omega_\mathrm{m}]\leftarrow\hat{c}_\mathrm{in}[\omega]}\hat{c}^\dagger_\mathrm{in}[\omega]\right) \\
& \left(S_{\hat{a}_\mathrm{out}[\omega+\omega_\mathrm{m}]\leftarrow\hat{a}_\mathrm{in}[\omega+\omega_\mathrm{m}]}\hat{a}_\mathrm{in}[\omega+\omega_\mathrm{m}]+S_{\hat{a}_\mathrm{out}[\omega+\omega_\mathrm{m}]\leftarrow\hat{c}_\mathrm{in}[\omega]}\hat{c}_\mathrm{in}[\omega]\right).
\end{aligned} \tag{A30}$$

To capture the noise properties of the optical output, one has to measure its symmetrized power spectral density

$$\begin{aligned}
\mathcal{S}_{\hat{a}_\mathrm{out}}[\omega+\omega_\mathrm{m}] =& \frac{1}{2}\left\langle\left\{\hat{a}_\mathrm{out}[\omega+\omega_\mathrm{m}],\hat{a}^\dagger_\mathrm{out}[\omega+\omega_\mathrm{m}]\right\}\right\rangle = \frac{1}{2}+\left\langle\hat{a}^\dagger_\mathrm{out}[\omega+\omega_\mathrm{m}]\hat{a}_\mathrm{out}[\omega+\omega_\mathrm{m}]\right\rangle \\
=& \eta_+[\omega]\left(\mathcal{S}_{\hat{c}_\mathrm{in}}[\omega]+\mathcal{S}^\uparrow_{\mathrm{added},+}[\omega]\right),
\end{aligned} \tag{A31}$$

where $\mathcal{S}_{\hat{c}_\mathrm{in}}[\omega]=\frac{1}{2}+\left\langle\hat{c}^\dagger_\mathrm{in}[\omega]\hat{c}_\mathrm{in}[\omega]\right\rangle$ is the power spectral density of the microwave photon flux at the input port. We define the on-chip photon number transduction efficiency as

$$\begin{aligned}
\eta_+[\omega] =& |S_{\hat{a}_\mathrm{out}[\omega+\omega_\mathrm{m}]\leftarrow\hat{c}_\mathrm{in}[\omega]}|^2 = |S_{\hat{c}_\mathrm{out}[\omega]\leftarrow\hat{a}_\mathrm{in}[\omega+\omega_\mathrm{m}]}|^2 \\
=& \frac{\kappa_{\mathrm{ex},+}\kappa_{\mathrm{ex,m}}|g_+|^2|\chi_+[\omega]|^2|\chi_\mathrm{m}[\omega]|^2}{|1+|g_+|^2\chi_+[\omega]\chi_\mathrm{m}[\omega]|^2} \approx \frac{\kappa_{\mathrm{ex},+}}{\kappa_+}\frac{\kappa_{\mathrm{ex,m}}}{\kappa_\mathrm{m}}\frac{4C_+}{(1+C_+)^2}
\end{aligned} \tag{A32}$$

and the added noise during up-conversion as

$$\mathcal{S}^\uparrow_{\mathrm{added},+}[\omega] = \frac{1}{2\eta_+[\omega]}\left(1-\eta_+[\omega]-|S_{\hat{a}_\mathrm{out}[\omega+\omega_\mathrm{m}]\leftarrow\hat{a}_\mathrm{in}[\omega+\omega_\mathrm{m}]}|^2\right)+\frac{|S_{\hat{a}_\mathrm{out}[\omega+\omega_\mathrm{m}]\leftarrow\hat{a}_\mathrm{in}[\omega+\omega_\mathrm{m}]}|^2}{\eta_+[\omega]}\mathcal{S}_{\hat{a}_\mathrm{in}}[\omega+\omega_\mathrm{m}]. \tag{A33}$$

We used the fact that the noise from the two input ports are not correlated, i.e. $\left\langle\hat{c}^\dagger_\mathrm{in}[\omega]\hat{a}_\mathrm{in}[\omega+\omega_\mathrm{m}]\right\rangle = \left\langle\hat{c}_\mathrm{in}[\omega]\hat{a}^\dagger_\mathrm{in}[\omega+\omega_\mathrm{m}]\right\rangle = 0$. An additional noise of $\frac{1}{2\eta_+[\omega]}\left(1-\eta_+[\omega]-|S_{\hat{a}_\mathrm{out}[\omega+\omega_\mathrm{m}]\leftarrow\hat{a}_\mathrm{in}[\omega+\omega_\mathrm{m}]}|^2\right)$ is introduced so that

the output channel still respect the bosonic commutation relations [3]. The last approximation of Eq. A32 gives the steady-state efficiency of the transducer ($\omega \to 0$), which depends on anti-Stokes cooperativity $C_+ = 4g_+^2/(\kappa_+\kappa_\mathrm{m})$ as well as extraction efficiencies of the antisymmetric supermode $\kappa_{\mathrm{ex},+}/\kappa_+$ and the acoustic mode $\kappa_{\mathrm{ex,m}}/\kappa_\mathrm{m}$. Similarly, the symmetrized power spectral density of the output photon number flux at the microwave port is

$$\mathcal{S}_{\hat{c}_\mathrm{out}}[\omega] = \frac{1}{2}\left\langle\left\{\hat{c}_\mathrm{out}[\omega], \hat{c}^\dagger_\mathrm{out}[\omega]\right\}\right\rangle = \frac{1}{2} + \left\langle\hat{c}^\dagger_\mathrm{out}[\omega]\hat{c}_\mathrm{out}[\omega]\right\rangle = \eta_+[\omega]\left(\mathcal{S}_{\hat{a}_\mathrm{in}}[\omega+\omega_\mathrm{m}] + \mathcal{S}^\downarrow_{\mathrm{added},+}[\omega]\right). \tag{A34}$$

While the expressions for the conversion efficiency are identical for both up- and down-conversion, the added noise for down-conversion is instead

$$\mathcal{S}^\downarrow_{\mathrm{added},+}[\omega] = \frac{1}{2\eta_+[\omega]}\left(1 - \eta_+[\omega] - |S_{\hat{c}_\mathrm{out}[\omega]\leftarrow\hat{c}_\mathrm{in}[\omega]}|^2\right) + \frac{|S_{\hat{c}_\mathrm{out}[\omega]\leftarrow\hat{c}_\mathrm{in}[\omega]}|^2}{\eta_+[\omega]}\mathcal{S}_{\hat{c}_\mathrm{in}}[\omega]. \tag{A35}$$

b. *Stokes configuration*

We follow the same recipe to derive the conversion efficiency and added noise for the Stokes configuration where $\Delta_- = \omega_\mathrm{m}$ and $\Delta_+ = 0$. We have the optical input-output relation

$$\hat{a}_\mathrm{out}[\omega] = \hat{a}_\mathrm{in}[\omega] - \sqrt{\kappa_{\mathrm{ex},-}}\hat{a}_-[\omega+\omega_\mathrm{m}] - \sqrt{\kappa_{\mathrm{ex},+}}\hat{a}_+[\omega] \tag{A36}$$

and the equations of motion

$$\begin{aligned}
\hat{a}_-[\omega] &= ig_-\chi_-[\omega]\left(\hat{b}[\omega-2\omega_\mathrm{m}] + \hat{b}^\dagger[\omega]\right) + \sqrt{\kappa_{\mathrm{ex},-}}\chi_-[\omega]\hat{a}_\mathrm{in}[\omega-\omega_\mathrm{m}]\\
&\approx ig_-\chi_-[\omega]\hat{b}^\dagger[\omega] + \sqrt{\kappa_{\mathrm{ex},-}}\chi_-[\omega]\hat{a}_\mathrm{in}[\omega-\omega_\mathrm{m}],\\
\hat{a}_+[\omega] &= ig_+\chi_+[\omega]\left(\hat{b}[\omega-\omega_\mathrm{m}] + \hat{b}^\dagger[\omega+\omega_\mathrm{m}]\right) + \sqrt{\kappa_{\mathrm{ex},+}}\chi_+[\omega]\hat{a}_\mathrm{in}[\omega]\\
&\approx \sqrt{\kappa_{\mathrm{ex},+}}\chi_+[\omega]\hat{a}_\mathrm{in}[\omega],\\
\hat{b}[\omega] &= ig_-^*\chi_\mathrm{m}[\omega]\hat{a}_-[\omega+2\omega_\mathrm{m}] + ig_-\chi_\mathrm{m}[\omega]\hat{a}_-^\dagger[\omega] + ig_+^*\chi_\mathrm{m}[\omega]\hat{a}_+[\omega+\omega_\mathrm{m}] + ig_+\chi_\mathrm{m}[\omega]\hat{a}_+^\dagger[\omega+\omega_\mathrm{m}] + \sqrt{\kappa_\mathrm{ex,m}}\chi_\mathrm{m}[\omega]\hat{c}_\mathrm{in}[\omega]\\
&\approx ig_-\chi_\mathrm{m}[\omega]\hat{a}_-^\dagger[\omega] + \sqrt{\kappa_\mathrm{ex,m}}\chi_\mathrm{m}[\omega]\hat{c}_\mathrm{in}[\omega].
\end{aligned} \tag{A37}$$

Using the signal flow graph shown in Supplementary Fig. 1b, the transfer functions between the microwave field at ω_m and the optical field at $\omega_\mathrm{L} - \omega_\mathrm{m}$ can once again be derived as

$$S_{\hat{a}_\mathrm{out}[\omega-\omega_\mathrm{m}]\leftarrow\hat{c}^\dagger_\mathrm{in}[\omega]} = S^*_{\hat{a}^\dagger_\mathrm{out}[\omega-\omega_\mathrm{m}]\leftarrow\hat{c}_\mathrm{in}[\omega]} = \frac{-i\sqrt{\kappa_{\mathrm{ex},-}}\sqrt{\kappa_\mathrm{ex,m}}g_-\chi_-[\omega]\chi^*_\mathrm{m}[\omega]}{1-|g_-|^2\chi^*_-[\omega]\chi_\mathrm{m}[\omega]}, \tag{A38}$$

$$S_{\hat{c}_\mathrm{out}[\omega]\leftarrow\hat{a}^\dagger_\mathrm{in}[\omega-\omega_\mathrm{m}]} = S^*_{\hat{c}^\dagger_\mathrm{out}[\omega]\leftarrow\hat{a}_\mathrm{in}[\omega-\omega_\mathrm{m}]} = \frac{i\sqrt{\kappa_{\mathrm{ex},-}}\sqrt{\kappa_\mathrm{ex,m}}g_-\chi^*_-[\omega]\chi_\mathrm{m}[\omega]}{1-|g_-|^2\chi_-[\omega]\chi^*_\mathrm{m}[\omega]}. \tag{A39}$$

While the microwave-microwave transfer function is identical to Eq. A29, the optical-optical transfer function is

$$S_{\hat{a}_\mathrm{out}[\omega-\omega_\mathrm{m}]\leftarrow\hat{a}_\mathrm{in}[\omega-\omega_\mathrm{m}]} = 1 - \frac{\kappa_{\mathrm{ex},-}\chi_-[\omega]}{1-|g_-|^2\chi^*_-[\omega]\chi_\mathrm{m}[\omega]} - \kappa_{\mathrm{ex},+}\chi_+[\omega-\omega_\mathrm{m}]. \tag{A40}$$

The photon number flux at the optical output of the transducer has a symmetrized power spectral density

$$\begin{aligned}
\mathcal{S}_{\hat{a}_\mathrm{out}}[\omega-\omega_\mathrm{m}] &= \frac{1}{2}\left\langle\left\{\hat{a}_\mathrm{out}[\omega-\omega_\mathrm{m}], \hat{a}^\dagger_\mathrm{out}[\omega-\omega_\mathrm{m}]\right\}\right\rangle = \frac{1}{2} + \left\langle\hat{a}^\dagger_\mathrm{out}[\omega-\omega_\mathrm{m}]\hat{a}_\mathrm{out}[\omega-\omega_\mathrm{m}]\right\rangle\\
&= \eta_-[\omega]\left(\mathcal{S}_{\hat{c}_\mathrm{in}}[\omega] + \mathcal{S}^\uparrow_{\mathrm{added},-}[\omega]\right).
\end{aligned} \tag{A41}$$

We define the on-chip photon number conversion efficiency for the Stokes configuration as

$$\begin{aligned}
\eta_-[\omega] &= |S_{\hat{a}_\mathrm{out}[\omega-\omega_\mathrm{m}]\leftarrow\hat{c}^\dagger_\mathrm{in}[\omega]}|^2 = |S_{\hat{c}_\mathrm{out}[\omega]\leftarrow\hat{a}^\dagger_\mathrm{in}[\omega-\omega_\mathrm{m}]}|^2\\
&= \frac{\kappa_{\mathrm{ex},-}\kappa_\mathrm{ex,m}|g_-|^2|\chi_-[\omega]|^2|\chi_\mathrm{m}[\omega]|^2}{|1+|g_-|^2\chi_-[\omega]\chi_\mathrm{m}[\omega]|^2} \approx \frac{\kappa_{\mathrm{ex},-}}{\kappa_-}\frac{\kappa_\mathrm{ex,m}}{\kappa_\mathrm{m}}\frac{4C_-}{(1-C_-)^2}
\end{aligned} \tag{A42}$$

as well as the added noise during up-conversion

$$\mathcal{S}^{\uparrow}_{\mathrm{added},-}[\omega] = \frac{1}{2\eta_-[\omega]}\left(1+\eta_-[\omega] - |S_{\hat{a}_{\mathrm{out}}[\omega-\omega_{\mathrm{m}}]\leftarrow \hat{a}_{\mathrm{in}}[\omega-\omega_{\mathrm{m}}]}|^2\right) + \frac{|S_{\hat{a}_{\mathrm{out}}[\omega-\omega_{\mathrm{m}}]\leftarrow \hat{a}_{\mathrm{in}}[\omega-\omega_{\mathrm{m}}]}|^2}{\eta_-[\omega]}\,\mathcal{S}_{\hat{a}_{\mathrm{in}}}[\omega-\omega_{\mathrm{m}}] \tag{A43}$$

where $C_- = 4g_-^2/(\kappa_-\kappa_{\mathrm{m}})$ is the Stokes cooperativity. The output photon number at the microwave port for the Stokes configuration is given by

$$\mathcal{S}_{\hat{c}_{\mathrm{out}}}[\omega] = \frac{1}{2}\left\langle\left\{\hat{c}_{\mathrm{out}}[\omega],\hat{c}^{\dagger}_{\mathrm{out}}[\omega]\right\}\right\rangle = \frac{1}{2}+\left\langle\hat{c}^{\dagger}_{\mathrm{out}}[\omega]\hat{c}_{\mathrm{out}}[\omega]\right\rangle = \eta_-[\omega]\left(\mathcal{S}_{\hat{a}_{\mathrm{in}}}[\omega-\omega_{\mathrm{m}}]+\mathcal{S}^{\downarrow}_{\mathrm{added},-}[\omega]\right). \tag{A44}$$

The photon number on-chip conversion efficiency is again the same for both up- and down-conversion, but the added noise for the down-conversion process is instead

$$\mathcal{S}^{\downarrow}_{\mathrm{added},-}[\omega] = \frac{1}{2\eta_-[\omega]}\left(1+\eta_-[\omega] - |S_{\hat{c}_{\mathrm{out}}[\omega]\leftarrow \hat{c}_{\mathrm{in}}[\omega]}|^2\right) + \frac{|S_{\hat{c}_{\mathrm{out}}[\omega]\leftarrow \hat{c}_{\mathrm{in}}[\omega]}|^2}{\eta_-[\omega]}\,\mathcal{S}_{\hat{c}_{\mathrm{in}}}[\omega]. \tag{A45}$$

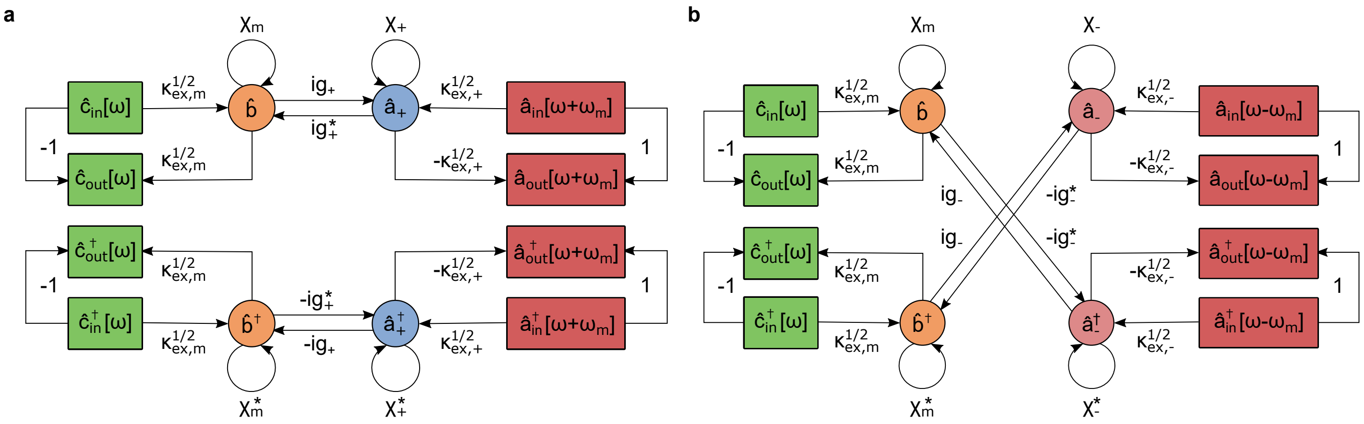

Supplementary Fig. 1. **Signal flow graphs of the frequency conversion processes.** **a** Effective beam-splitter process. **b** Effective two-mode-squeezing process. The bath operators are indicated by rectangles, the internal modes of the transducers by circles and the coupling rates by arrows.

4. Estimation of correlated microwave-optical pair generation rates

Equations A41 and A44 suggest that entangled pairs of microwave and optical photons can be generated in the Stokes configuration since it gives rise to an effective two-mode squeezing Hamiltonian [4–6]. In order to verify that the HBAR transducer can generate entangled pairs of microwave and optical photons, we compute the on-chip pair generation rate, expected to be equal to the output photon flux from the lower frequency optical mode as in standard spontaneous parametric down-conversion (SPDC) scheme. Integrating over the transduction bandwidth, we have

$$R = \int_{-\infty}^{\infty}\left\langle\hat{a}^{\dagger}_{\mathrm{out}}[\omega]\hat{a}_{\mathrm{out}}[\omega]\right\rangle d\omega \approx \int_{-\infty}^{\infty}\eta_-[\omega]d\omega \approx \frac{\pi}{2}\left(\kappa_-^{-1}+\kappa_{\mathrm{m}}^{-1}\right)^{-1}\eta_-[0]. \tag{A46}$$

In practice, the pump laser should be filtered for the optical output to be dominated by heralding photons, and other losses from the measurement setup can further reduce the effective rate. For $\eta^{\mathrm{oc}} = 7.9\times10^{-5}$, $\kappa_- = 2\pi\times 166\,\mathrm{MHz}$ and $\kappa_{\mathrm{m}} = 2\pi\times 13\,\mathrm{MHz}$, this would corresponds to an on-chip pair generation rate of $R \approx 2\pi\times 1.5\,\mathrm{kHz}$. Losses in the measurement chain have to be accounted for to estimate the count rate at the detector. In order to measure entanglement between the microwave and optical fields, the on-chip rate must be higher than the thermal decoherence rate of the acoustic mode $\Gamma^{\mathrm{dec}} = \kappa_{\mathrm{m}} n_{\mathrm{th,m}}(\omega_{\mathrm{m}})$, where the thermal occupancy is given by the Bose-Einstein distribution $n_{\mathrm{th,m}}(\omega) = \left[\exp\left(\hbar\omega/(k_{\mathrm{B}}T)\right)-1\right]^{-1}$ given a temperature T and Boltzmann's constant k_{B}. For $\omega_{\mathrm{m}} = 2\pi\times 3.5\,\mathrm{GHz}$ and $\kappa_{\mathrm{m}} = 2\pi\times 13\,\mathrm{MHz}$, the decoherence rate $\Gamma^{\mathrm{dec}}/(2\pi)$ at $800\,\mathrm{mK}$ is $56\,\mathrm{MHz}$ and $0.7\,\mathrm{Hz}$ at $10\,\mathrm{mK}$. Therefore, the transducer needs to be operated in the mixing chamber of the dilution refrigerator, with a pulsed pump to reduce heat load. The total rate of optical heralding is hence gated by the pump duty cycle.

To evaluate the non-classical correlation required for the DLCZ protocol [6, 7], one can compute the second-order cross-correlation function. Since no input signal needs to be sent to the transducer for SPDC, $\langle \hat{a}_{\rm in} \rangle = \left\langle \hat{a}^\dagger_{\rm in} \right\rangle = \left\langle \hat{a}^\dagger_{\rm in}\hat{a}_{\rm in} \right\rangle = \langle \hat{a}_{\rm in}\hat{a}_{\rm in} \rangle = \left\langle \hat{a}^\dagger_{\rm in}\hat{a}^\dagger_{\rm in} \right\rangle = 0$ and $\left\langle \hat{c}^\dagger_{\rm in}[\omega]\hat{c}_{\rm in}[\omega] \right\rangle = n_{\rm th,m}(\omega)$, leading to

$$\begin{aligned} g^{(2)}_{ac} =& \frac{\left\langle \hat{c}^\dagger_{\rm out}\hat{a}^\dagger_{\rm out}\hat{a}_{\rm out}\hat{c}_{\rm out} \right\rangle}{\left\langle \hat{c}^\dagger_{\rm out}\hat{c}_{\rm out} \right\rangle \left\langle \hat{a}^\dagger_{\rm out}\hat{a}_{\rm out} \right\rangle} \\ \approx& \frac{\left(|S_{\hat{a}_{\rm out}\leftarrow\hat{a}_{\rm in}}|^2 + \eta_-\right)}{\left(\eta_- + |S_{\hat{c}_{\rm out}\leftarrow\hat{c}_{\rm in}}|^2 n_{\rm th,m}\right)\left(1+n_{\rm th,m}\right)} + \frac{n_{\rm th,m}}{1+n_{\rm th,m}} \\ &+ \frac{\left(S^*_{\hat{a}_{\rm out}\leftarrow\hat{a}_{\rm in}} S_{\hat{a}_{\rm out}\leftarrow\hat{c}^\dagger_{\rm in}} S^*_{\hat{c}_{\rm out}\leftarrow\hat{a}^\dagger_{\rm in}} S_{\hat{c}_{\rm out}\leftarrow\hat{c}_{\rm in}} + S_{\hat{a}_{\rm out}\leftarrow\hat{a}_{\rm in}} S^*_{\hat{a}_{\rm out}\leftarrow\hat{c}^\dagger_{\rm in}} S_{\hat{c}_{\rm out}\leftarrow\hat{a}^\dagger_{\rm in}} S^*_{\hat{c}_{\rm out}\leftarrow\hat{c}_{\rm in}}\right) n_{\rm th,m}}{\eta_-\left(\eta_- + |S_{\hat{c}_{\rm out}\leftarrow\hat{c}_{\rm in}}|^2 n_{\rm th,m}\right)\left(1+n_{\rm th,m}\right)} \end{aligned} \tag{A47}$$

where the frequency dependences are omitted for readability. Non-classicality is expected to lead to violation of the Cauchy-Schwarz inequality, given by $g^{(2)}_{ac} \leq \sqrt{g^{(2)}_{aa} g^{(2)}_{cc}}$. Therefore, it is desirable to minimize both microwave thermal occupancy (cooling) and optical transmission (attaining critical coupling).

Appendix B: Fabrication process flow

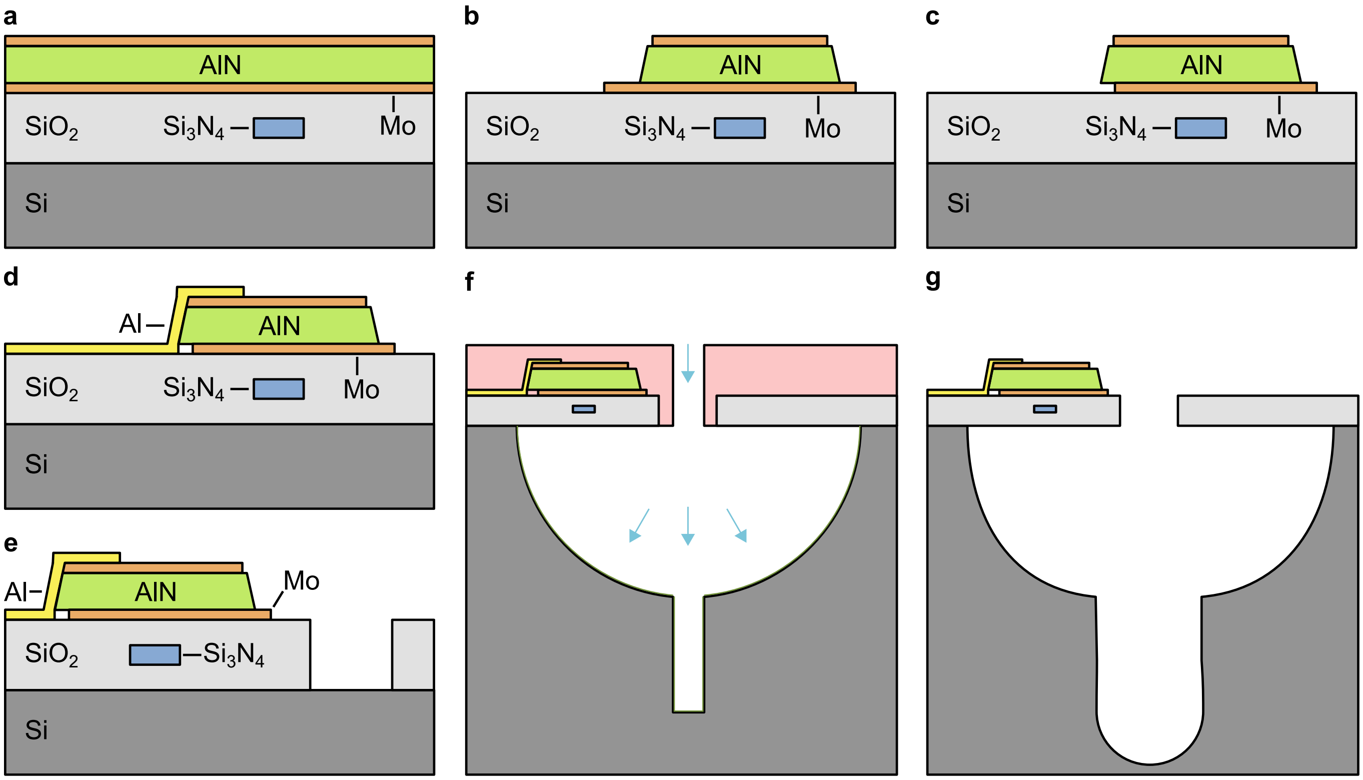

Supplementary Fig. 2. **Process flow for the fabrication of piezoelectric actuators on top of the photonic circuitry.** **a** Sputtering of the metal and piezoelectric layers. **b** Patterning of the top metal, piezoelectric and bottom metal layers. **c** XeF_2 etching of the bottom metal to avoid shorting the electrodes. **d** Sputtering of a second top metal layer to connect the top of the actuators to a feedline. **e** Dry etching of the oxide cladding to define the chip facets and the actuator holes. **f** Dry etching of the silicon substrate to suspend the actuators. **g** Deep reactive ion etching of the substrate to facilitate chip release.

The piezoelectric actuators are monolithically integrated on Si_3N_4 waveguides, fabricated using the photonic Damascene process [8, 9]. The 850-nm-thick Si_3N_4 film is deposited using low-pressure chemical vapor deposition into

the Damascene preform, with 2.8 µm thermal oxide below the waveguides. After annealing to drive hydrogen impurities out of the Si_3N_4 layer, 2.1-µm-thick TEOS oxide and 1.0-µm-thick low-temperature oxide top claddings are deposited and subsequently annealed. The metallic and piezoelectric films—95 nm of Mo, 1.0 µm of AlN and another 95 nm of Mo—are sputtered through foundry services provided by Plasma-Therm [10]. They are then patterned using chlorine-based reactive ion etching to form the actuators, ground planes and integrated heaters. We employ the same Band-Aid process as in Ref. [11] to localize the electrodes atop the suspended cladding. In particular, when connecting the Al feedline to the top electrode by a lift-off process, the bottom electrode is etched back using XeF_2 to avoid short-circuiting the electrodes. The process to suspend the cladding in the vicinity of the actuator is combined with the chip singulation steps of the Damascene process. First, a hole is opened in the middle of the arc-shaped actuator. The chip facets are simultaneously defined where trenches in the SiO_2 are etched by C_4F_8 deep reactive ion etching (DRIE) between neighboring chips. A second photolithography is performed to protect the facets while leaving the etched holes exposed for further processing, resulting in narrower trenches between chips. The Si substrate is then isotropically etched with SF_6 until the SiO_2 cladding below the actuator is suspended [12]. Following cladding suspension, the Si substrate is etched anisotropically until the desired chip thickness of 250 µm is reached. A final isotropic Si etch removes the parts of the substrate protruding from the SiO_2 facet, providing proper access to the bus waveguide nanotapers with lensed fibers, or even using the butt-coupling scheme. To facilitate this last step, we employ ion-beam etching at a 20° tilt angle to remove the passivation layer formed on the hole sidewalls during the Si DRIE. The chips are ultimately released by grinding the back side of the wafer. This convoluted process, summarized in Supplementary Fig. 2, ensures that all the actuators on the wafer are fully suspended, while the chip facets do not become exceedingly fragile from the undercut.

Appendix C: Optical characterization

1. Resonance linewidths

Fitting the hybridized transmission spectrum in Fig. 2c to the coupled mode theory presented in Appendix A 1 and in Ref. [2] yields $\kappa_\mathrm{r}/(2\pi) = 154\,\mathrm{MHz}$ and $\kappa_\mathrm{l}/(2\pi) = 190\,\mathrm{MHz}$, with $\kappa_\mathrm{ex,l}/(2\pi) = \kappa_\mathrm{ex,r}/(2\pi) = 60\,\mathrm{MHz}$.

2. Hybridization of optical resonances via integrated thermo-optic heaters

For the purpose of rapid characterization of the photonic molecules at room temperature, the bottom electrode of the piezoelectric actuator also serves as an integrated heater to control the relative detuning between the microrings by thermo-optic effect. As seen in Fig. 2a, we pattern Mo to make three electrical connections to the bottom electrode. The top and bottom connections are connected to ground, whereas the central connection is biased to a constant voltage. The finite resistance of Mo at room temperature leads to Joule heating as current flows through the bottom electrode, modifying the refractive index of the Si_3N_4 waveguide buried directly underneath. The heaters were only used for fast search of devices with adequate mode splitting.

3. Thermal response characterization through cavity-enhanced photothermal spectroscopy

For the purpose of programming the pulse sequence to alleviate thermal effects, we study the thermal response of the present transducer due to optical pump heating. Specifically, thermally induced refractive index change causes a shift in resonance frequency of the Si_3N_4 micro-ring, which is detected using cavity-enhanced pump-probe spectroscopy [13]. The intensity-modulated pump addresses a TE resonance, while the probe measures the resulting side-of-fringe modulation of another TE mode. To ascertain the physical mechanism of each cross phase modulation (XPM) process, we sweep the pump modulation frequency to leverage the separation of time scales. Seen in Supplementary Fig. 3, the response exhibits three plateaus. We associate the process at modulation frequencies $\gtrsim 1$ MHz with Kerr-induced XPM. The two slower processes at ~ 100 kHz and ~ 1 kHz are characteristic of photothermal XPM in quasi-free-standing microresonators such as spheres [14] and toroids [15]. Here, the former "local" time scale can be attributed to thermalization of the mode volume with the acoustic resonator, while the thermalization of the suspended structure with the rest of the chip constitutes the latter "global" time scale. Therefore, it is expected that for future cryogenic operations, employing a pulse-on time (τ_on) shorter than the local time scale to gate the optical pump will significantly reduce thermal occupancy in the cladding acoustic mode. The pulse repetition rate (f_rep) and hence pulse-off time can then be chosen based on the available cooling power.

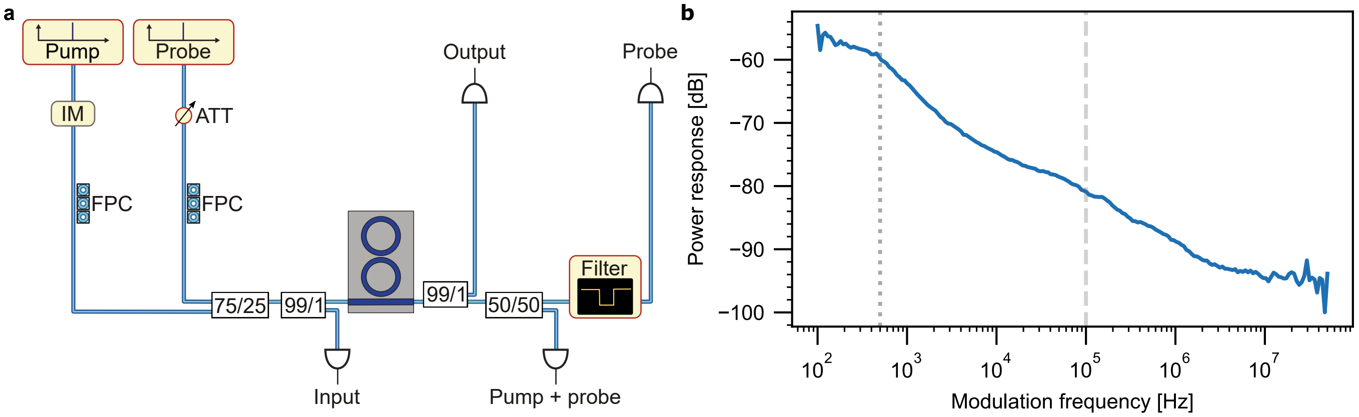

Supplementary Fig. 3. **Thermal response measurement through pump-probe spectroscopy. a** Schematic of the setup used for optical pump-probe spectroscopy. A pump laser, addressing a micro-ring resonance and modulated by an intensity modulator (IM), induces photothermal and Kerr cross-phase modulations. As a result, the frequency of a nearby resonance is also modulated, which is detected by a probe laser. The power of the probe laser is adjusted by a variable attenuator (ATT). The pump and probe polarizations are optimized with their respective fiber polarization controllers (FPC). The white boxes represent fiber beam splitters. **b** Measured cross-phase modulation response of a micro-ring as detected at the "Probe" photodetector with the pump filtered. The dotted and dashed lines indicate the 3-dB response bandwidths of the "global" and "local" photothermal processes, respectively.

Appendix D: Characterization of acoustic resonances

The microwave reflection measurements (also known as S_{11}) shown here were measured at room temperature using custom probes (GGB industries nickel-alloy 40A-GS-135-PC-N Picoprobe) and a vector network analyzer (Rhode&Schwarz ZNB-20). The spectrum shown in Fig. 2d is corrected using a calibration substrate (GGB industries CS-8) to remove the phase delay and attenuation introduced by coaxial cables and probes. However, this calibration procedure was only used to get the S-parameter, and was not applied during the total conversion efficiency measurements shown in Figs. 3 and 4. In a previous version of the transducers and in Ref. [12] where the Band-Aid process (Appendix B) is not employed, the piezoelectric actuator extends beyond the suspended cladding and covers the electrical feedline, causing two detrimental effects for transduction. First, the resulting stray capacitance leads to excess microwave insertion loss. Given the small size of the suspended area ($\sim 1\,000\,\mu\mathrm{m}^2$) compared to that of the feedline ($\gtrsim 10\,000\,\mu\mathrm{m}^2$), this effect is readily seen in Supplementary Fig. 4, where the spectrum without the Band-Aid process exhibits reduced background reflection. Second, the part of the actuator on top of the unreleased cladding leads to resonant excitation of HBAR modes extending over the entire Si substrate. These modes have a smaller free spectral range (FSR) of around 19 MHz and a lower microwave extraction efficiency. The theory presented in Appendix A does not correspond to the case of such a multimode system as it assumes being in the sideband-resolved regime, implying the high density of substrate modes is inappropriate for low-noise frequency conversion. The feedline Band-Aid, discussed in Appendix B and shown in Supplementary Fig. 4, enables the removal of the piezoelectric layer above the substrate, leaving only HBAR modes well confined in the oxide cladding.

We fit the measured complex reflection to obtain the mechanical quality factor $Q_\mathrm{m} = 284$ and microwave extraction efficiency $\kappa_\mathrm{ex,m}/\kappa_\mathrm{m} = 0.11$ for the transduction mode [16]. This fit model comprises multiple Fano resonances, given by

$$S_\mathrm{EE}[\omega] = Ae^{i\alpha - i\omega\tau}\left(1 - \sum_r e^{i\phi_r}\frac{2Q_\mathrm{l}^r/Q_\mathrm{c}^r}{1 + 2iQ_\mathrm{l}^r(\omega - \omega_r)/\omega_r}\right) \tag{D1}$$

and the result is shown in Supplementary Fig. 5a. In addition, we simulate the microwave reflection of the designed stack geometry using finite-element method (FEM). Fitting the simulated spectrum leads to $Q_\mathrm{m} = 205$ and $\kappa_\mathrm{ex,m}/\kappa_\mathrm{m} = 0.61$. The discrepancy is attributed to difference in clamping losses and piezoelectric coefficients between the fabricated and simulated devices that lift the degeneracy of electromechanical modes. We extract the effective mass of the transduction HBAR mode from FEM simulations as

$$m_\mathrm{eff} = \frac{1}{\max(|\mathbf{x}(\mathbf{r})|^2)}\iiint \rho(\mathbf{r})\mathbf{x}^*(\mathbf{r})\mathbf{x}(\mathbf{r})d\mathcal{V} \approx 6\,\mathrm{ng}, \tag{D2}$$

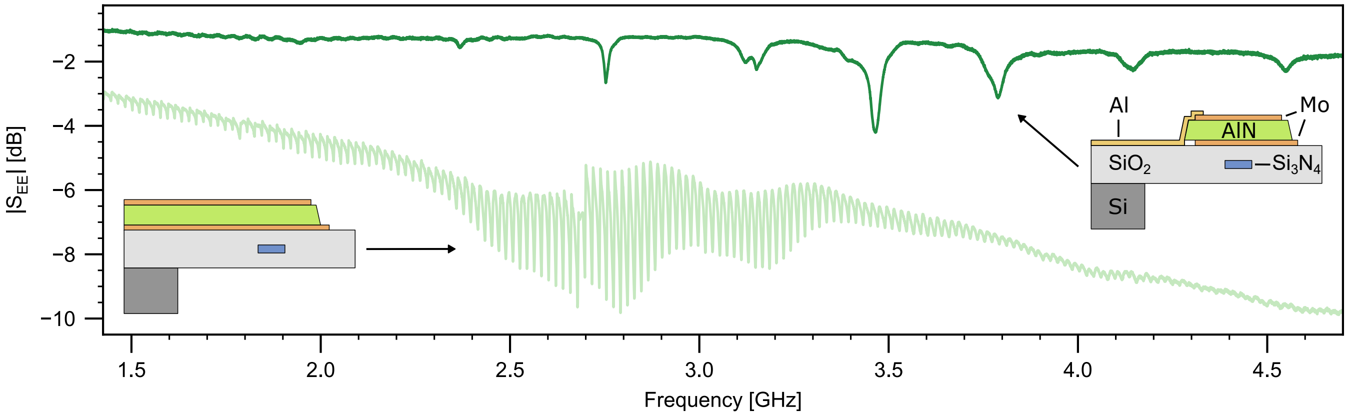

Supplementary Fig. 4. **Elimination of substrate HBAR modes.** Microwave reflection spectra obtained with and without applying the Band-Aid process. The respective stack compositions are indicated as insets.

where $\rho(\mathbf{r})$ is the local mass density and $\mathbf{x}(\mathbf{r})$ is the mechanical displacement, corresponding to $x_{\mathrm{ZPF}} = \sqrt{\hbar/(2m_{\mathrm{eff}}\omega_{\mathrm{m}})} \approx 2 \times 10^{-8}\,\mathrm{nm}$.

The admittance is obtained by fitting the microwave reflection measurement to $Y[\omega] = Z_0^{-1}\,(1 - S_{\mathrm{EE}}[\omega])\,/\,(1 + S_{\mathrm{EE}}[\omega])$, Z_0 being the characteristic impedance of the transmission line. Fitting the admittance to a modified Butterworth–van Dyke model yields a static capacitance of around 200 fF [17]. The small static capacitance is advantageous for reducing microwave insertion loss but comes at the cost of a reduced electromechanical transduction factor. Suspending the cladding decreases the acoustic mode volume, thereby enhancing the electromechanical coupling to the microwave port by improving the confinement in the piezoelectric layer.

Resonance	$\omega/(2\pi)$ [GHz]	$\kappa/(2\pi)$ [MHz]	$\kappa_{\mathrm{ex}}/\kappa$ [%]	ϕ [°]
1	3.479	13	11	5
2	3.495	27	6	5
3	3.463	28	6	11
4	3.440	40	4	−11
5	3.397	38	2	−11

Supplementary Table I. Parameters for the Fano resonance fit of the microwave reflection corresponding to Eq. D1.

Appendix E: Characterization of bidirectional transduction

1. Non-Lorentzian transduction bandwidth

For an ideal transducer, the transduction lineshape is expected to be Lorentzian in the low-cooperativity limit. Nevertheless, the measured lineshape (Figs. 3b and 3d) is evidently non-Lorentzian. We attribute this deviation to the presence of a second mechanical mode contributing to the transduction process. Supplementary Figure 5b shows the normalized susceptibilities of the principal mechanical mode, the auxiliary mechanical mode as well as the susceptibility of the higher frequency optical mode, considering that the device is operated in the anti-Stokes configuration. Supplementary Figure 5c illustrates the transduction spectrum that would be obtained from the theory exposed in Appendix A considering an additional mode and compares it with the experimental data shown in Fig. 3b. The only free parameters for the fit are the vacuum optomechanical coupling rates ($g_0^1 \approx 2\pi \times 20\,\mathrm{Hz}$ and $g_0^2 \approx 2\pi \times 35\,\mathrm{Hz}$), the other values being the same as in Appendix E 2. The difference with the vacuum optomechanical coupling rate estimated in Appendix E 2 comes from the off-resonant contribution of the second mechanical mode.

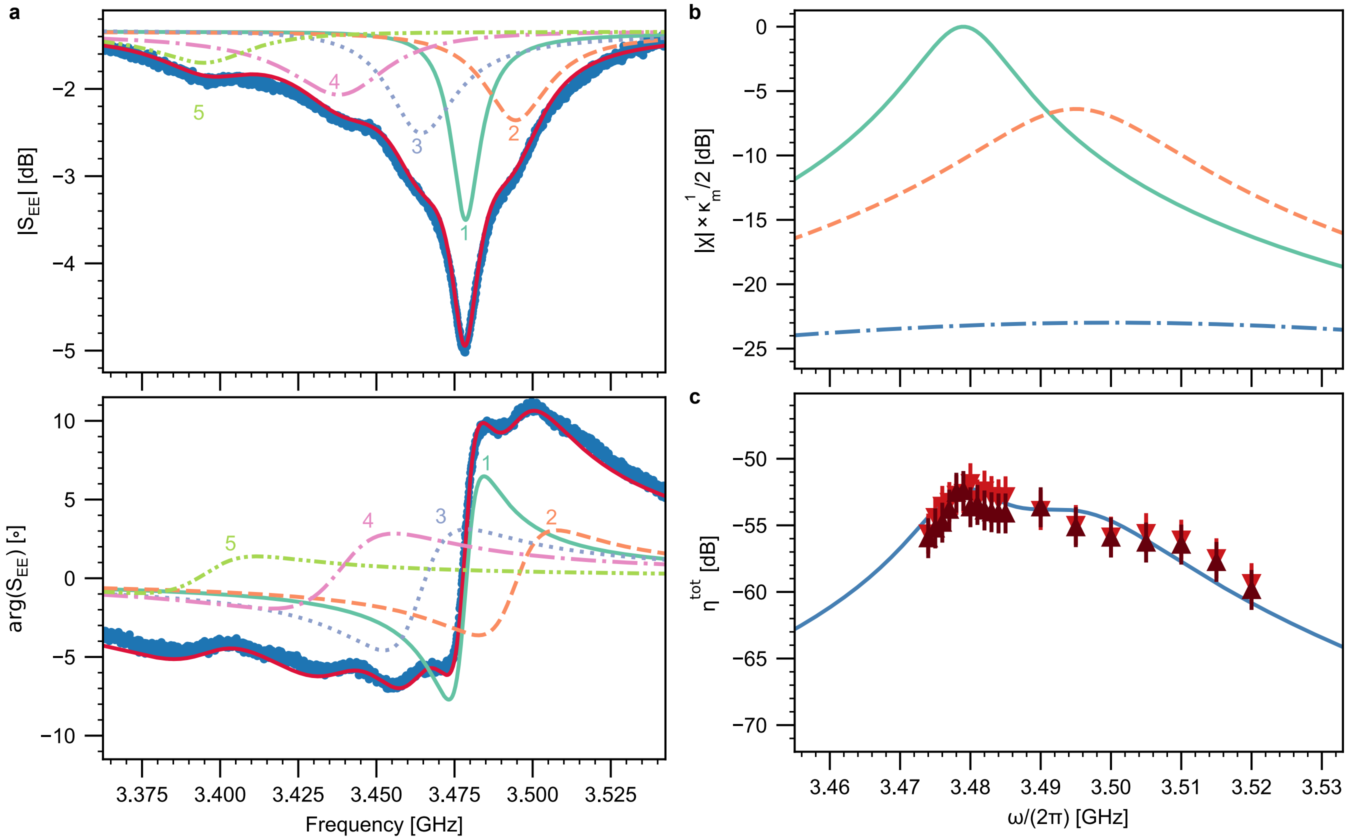

Supplementary Fig. 5. **Spurious acoustic modes near the main resonance. a** Fit of the measured microwave reflection using multiple Fano resonances. The top panel shows the amplitude of the reflection, while the bottom panel depicts its phase after removing the background electrical delay from the cables. The experimental data is shown in blue, the total fit red, and the response of single Fano resonances in the other numbered curves. The parameters used for the fit are indicated in Supplementary Table I. **b** Susceptibilities of the two acoustic resonances relevant for transduction (solid line for the main resonance, dashed line for the secondary mode), as well as the higher frequency optical mode (dashed-dotted line). The absolute value is normalized to the peak of the main acoustic resonance at 3.479 GHz. **c** Simulated total transduction efficiency in the anti-Stokes configuration using the two acoustic resonances from **b**. The triangles correspond to the experimental data from Fig. 3b. The vacuum optomechanical coupling rates are the only free parameters of this fit, which gives $g_0^1 \approx 2\pi \times 20\,\text{Hz}$ and $g_0^2 \approx 2\pi \times 35\,\text{Hz}$.

2. Estimation of cooperavity and vacuum coupling rate

The up- and down-conversion spectra, described in the main text and illustrated in Supplementary Fig. 6, provide the off-chip efficiency from which the vacuum coupling rate can be estimated. The off-chip efficiency is given by

$$\eta^{\text{tot}} = \eta^{\text{probes}}\eta^{\text{fiber-chip}}\eta^{\text{oc}}, \tag{E1}$$

where η_{probes} and $\eta_{\text{fiber-chip}}$ denote the microwave and optical fiber to chip insertion efficiencies, respectively. The on-chip efficiency can be written as

$$\eta^{\text{oc}} = \eta^{\text{ext}}\eta^{\text{int}} = \eta_{\text{o}}\eta_{\text{m}}\eta^{\text{int}}. \tag{E2}$$

The internal efficiency

$$\eta^{\text{int}} = \frac{4C}{(1 \pm C)^2} \approx 4C \tag{E3}$$

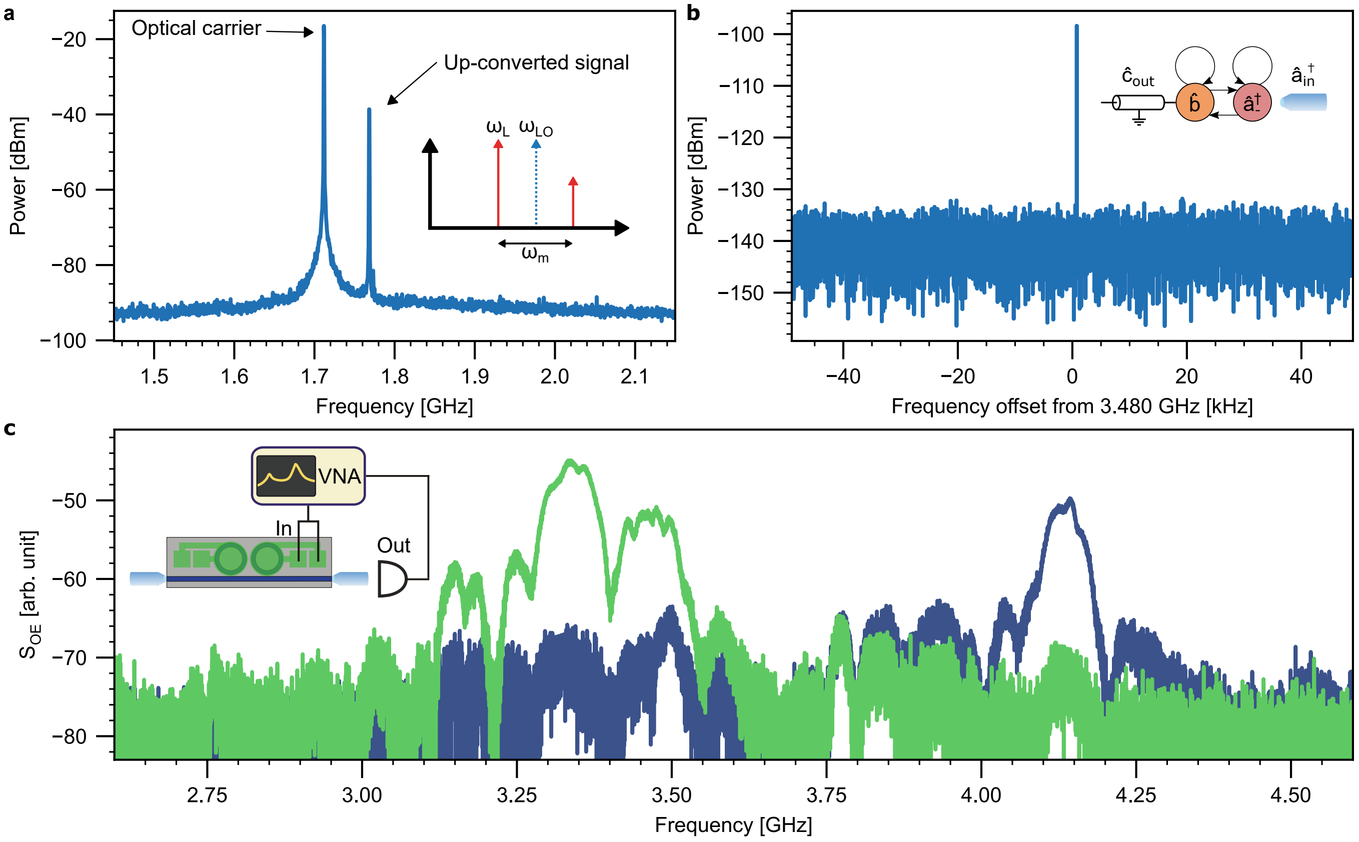

Supplementary Fig. 6. **Experimental dataset examples.** **a** Optical heterodyne spectrum for a local oscillator set between the carrier and the up-converted sideband, as indicated in the inset. **b** Microwave probes output measured with an electronic spectrum analyzer. The input optical spectrum contains both the carrier and sidebands generated by an external electro-optic modulator. In this example, the modulation frequency is set to 3.480 GHz. **c** Acousto-optic response for different laser-cavity detunings on the same transducer.

in the low-cooperativity regime, where the plus and minus signs correspond to the anti-Stokes and Stokes configurations, respectively. The optical and microwave extraction efficiencies

$$\eta_\mathrm{o} = \frac{\kappa_\mathrm{ex,o}}{\kappa_\mathrm{o}}, \tag{E4}$$

$$\eta_\mathrm{m} = \frac{\kappa_\mathrm{ex,m}}{\kappa_\mathrm{m}}. \tag{E5}$$

The single-photon cooperativity C_0 is enhanced by the intracavity photons

$$C = C_0 \bar{n}, \tag{E6}$$

$$\begin{aligned} n_c &= \kappa_\mathrm{ex} |\chi_\mathrm{o}|^2 \frac{\eta^{\mathrm{fiber-chip}} P_\mathrm{in}}{\hbar\omega_\mathrm{L}} \\ &\approx \frac{4\kappa_\mathrm{ex}}{\kappa_\mathrm{o}^2} \frac{\eta^{\mathrm{fiber-chip}} P_\mathrm{in}}{\hbar\omega_\mathrm{L}} = \eta_\mathrm{o} \frac{4}{\kappa_\mathrm{o}} \frac{\eta^{\mathrm{fiber-chip}} P_\mathrm{in}}{\hbar\omega_\mathrm{L}}, \end{aligned} \tag{E7}$$

where the pump detuning is assumed to be small. The off-chip efficiency with respect to the microwave probe and optical fiber is thus

$$\begin{aligned} \eta^\mathrm{tot} &\approx \eta^\mathrm{probes} \eta^\text{fiber-chip} \times \eta_\mathrm{m}\eta_\mathrm{o} \times 4C_0 \times \eta_\mathrm{o} \frac{4}{\kappa_\mathrm{o}} \frac{1}{\hbar\omega_\mathrm{L}} \eta^\text{fiber-chip} P_\mathrm{in} \\ &= 16 \eta^\mathrm{probes} \eta^\text{fiber-fiber} \times \eta_\mathrm{m} \eta_\mathrm{o}^2 \times \frac{C_0 P_\mathrm{in}}{\hbar\omega_\mathrm{L}\kappa_\mathrm{o}}, \end{aligned} \tag{E8}$$

where the fiber-fiber coupling efficiency $\eta^{\text{fiber-fiber}} = \eta^{\text{fiber-chip}} \times \eta^{\text{fiber-chip}}$. Calibration of the RF probes informed about the probes collections efficiency $\eta_{\text{probes}} \approx -3\,\text{dB}$. A fraction of light is tapped just before and after the coupling lensed fibers for power monitoring. We obtain a typical $\eta^{\text{fiber-fiber}} = -8\,\text{dB}$ and hence $\eta^{\text{fiber-chip}} = -4\,\text{dB}$. Fitting the microwave (optical) reflection spectrum yields $\eta_{\text{m}} = 11\%$ ($\eta_{\text{o}} = 35\%$). Given that the measured efficiency $\eta^{\text{tot}} = -60\,\text{dB}$ at $P_{\text{in}} = 10\,\text{dBm}$, it follows that the single-photon cooperativity is

$$C_0 = \frac{\eta^{\text{tot}} \times \hbar\omega_{\text{L}}\kappa_{\text{o}}}{16\eta^{\text{probes}}\eta^{\text{fiber-fiber}} \times \eta_{\text{m}}\eta_{\text{o}}^2 \times P_{\text{in}}} \approx 8 \times 10^{-13}. \tag{E9}$$

The vacuum coupling rate between the acoustic and bare optical modes is then

$$g_0 = 2 \times \sqrt{\frac{\kappa_{\text{o}}\kappa_{\text{m}}C_0}{4}} \approx 2\pi \times 42\,\text{Hz}. \tag{E10}$$

Here $\kappa_{\text{o}} = 2\pi \times 170\,\text{MHz}$, $\kappa_{\text{m}} = 2\pi \times 13\,\text{MHz}$ and the additional factor of two comes from the hybridization of the optical modes (Eq. A16).

3. Estimation of on-chip and internal transduction efficiencies

We have achieved $\eta^{\text{tot}} = -48\,\text{dB}$ at an input pump power of 21 dBm. With the losses quoted in Appendix E 2, the on-chip and internal efficiencies can be estimated. For conversion in either directions, the signal goes through the respective input and output ports exactly once, acquiring an attenuation of $\eta^{\text{probes}}\eta^{\text{fiber-chip}} = -7\,\text{dB}$. We therefore have $\eta^{\text{oc}} = 7.9 \times 10^{-5}$. Knowing the extraction efficiencies $\eta_{\text{o}} = 35\%$ and $\eta_{\text{m}} = 11\%$, we further obtain $\eta^{\text{int}} = 2 \times 10^{-3}$.

Appendix F: Optimization of optical extraction efficiency

An optimal optical external coupling can be chosen to maximize the total conversion efficiency. Equation E8 can be rewritten in terms of quality factors by using the relation $\kappa = \omega/Q$, which yields

$$\eta^{\text{tot}} \approx 16\eta^{\text{probes}}\eta^{\text{fiber-fiber}} \times \eta_{\text{m}}\eta_{\text{o}}^2 \times g_0^2 \frac{Q_{\text{m}}}{\omega_{\text{m}}}\frac{Q_{\text{o}}}{\omega_{\text{o}}} \times \frac{Q_{\text{o}}}{\omega_{\text{o}}}\frac{P_{\text{in}}}{\hbar\omega_{\text{L}}}. \tag{F1}$$

For the optics, we have $Q_{\text{o}} = Q_{\text{int}} + Q_{\text{ext}}$, where we separate the intrinsic quality factor Q_{int}, oftentimes fabrication-limited and hence not easily adjustable, from the external coupling quality factor Q_{ex} that can be readily engineered through coupler design. The optical extraction efficiency can thus be written in the form

$$\eta_{\text{o}} = \frac{\kappa_{\text{ex}}}{\kappa_{\text{ex}} + \kappa_0} = \frac{Q_{\text{int}}}{Q_{\text{ex}}}\left(1 + \frac{Q_{\text{int}}}{Q_{\text{ex}}}\right)^{-1} = \frac{R}{1+R}, \tag{F2}$$

where $R = Q_{\text{int}}/Q_{\text{ex}}$ and the total optical quality factor

$$Q_{\text{o}} = \frac{1}{Q_{\text{int}}^{-1} + Q_{\text{ex}}^{-1}} = Q_{\text{int}}\frac{1}{1+R}. \tag{F3}$$

The total efficiency in the low-cooperativity regime is then given by

$$\eta^{\text{tot}} \approx F\frac{R^2}{(1+R)^4}, \tag{F4}$$

where

$$F = 16\eta^{\text{probes}}\eta^{\text{fiber-fiber}} \times \eta_{\text{m}}g_0^2\frac{Q_{\text{m}}Q_{\text{int}}^2}{\omega_{\text{m}}\omega_{\text{o}}^2} \times \frac{P_{\text{in}}}{\hbar\omega_{\text{L}}}. \tag{F5}$$

For a given F, the optimal efficiency is achieved at the critical coupling condition $Q_{\text{ex}} = Q_{\text{int}}$, or $R = 1$.

Appendix G: Experimental setup and data acquisition

We employ two Toptica CTL 1550 external cavity diode lasers in our experimental setup, depicted in Supplementary Fig. 7. The first laser ("science laser"), amplified by an erbium-doped fiber amplifier, is used to pump the device and for generating the optical signal for down-conversion, whereas the second laser serves as the local oscillator for optical heterodyne detection. The detuning between the two lasers is monitored from their beat note on a photodetector fed to an electronic spectrum analyzer. Optical transmission spectra are acquired using an oscilloscope while the science laser is scanned slowly. Their frequency axis is calibrated using a Mach-Zehnder interferometer with a long delay line in one arm. Optical powermeters are inserted before and after the chip, while a variable optical attenuator is used to control the power at the chip input. Fiber polarization controllers are used to ensure the science laser address the TE modes of the microresonators.

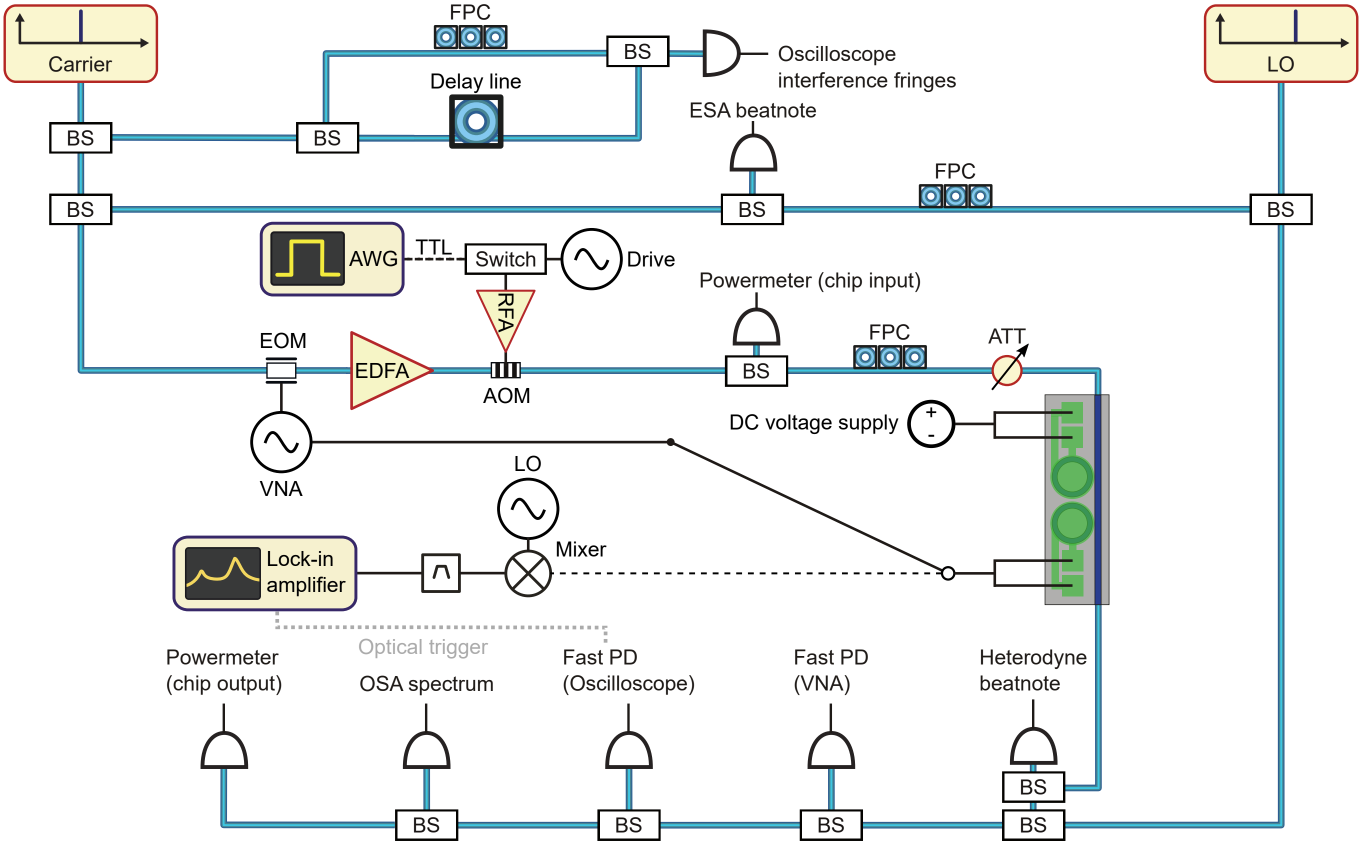

Supplementary Fig. 7. **Experimental setup.** The setup to measure the conversion efficiency is divided in three parts. First, part of the laser light is picked to calibrate its frequency using a Mach-Zehnder interferometer where a long delay line is introduced in one of the arm. The second laser, which provide the local oscillator, is beaten with the science laser for frequency calibration of the heterodyne signal. Finally, the science laser goes through the chip. Powermeters are placed before and after the chip in order to measure the insertion loss. The vector network analyzer (VNA) is used during the CW measurements, either for generating optical sidebands or for exciting the mechanics. During the pulsed measurements, the VNA is only used during up-conversion, being replaced by the lock-in detection for the down-conversion. Abbreviations used: AOM: acousto-optic modulator; ATT: variable optical attenuator; AWG: arbitrary waveform generator; BS: beam-splitter; EDFA: erbium-doped fiber amplifier; EOM: electro-optic modulator; FPC: fiber polarization controller; LO: local oscillator; OSA: optical spectrum analyzer; PD: photodetector; RFA: radio frequency amplifier; TTL: transistor-transistor logic.

For resonant transduction, the laser is tuned redward into the optical resonance from the blue side [18]. The laser-cavity detuning is chosen to maximize the acousto-optic response, monitored on a vector network analyzer. Regarding the acousto-optic response, one sideband is absorbed by the optical cavity, while the beating between the unsuppressed sideband and the carrier on a photodiode provides the signal. For driving and probing the HBAR, the piezoelectric actuator on the chip are connected to high-frequency RF probes (Picoprobe model 40A from GGB industries). We observed that the microwave reflection spectrum remains stable over the time scale of several months, provided the probe contact condition are maintained. We did not observe any change in resonance or broadening within the

range of microwave probe power up to 13 dBm and optical pump power up to 25 dBm. For the down-conversion demonstration, an external electro-optic modulator creates sidebands on the science laser. Their amplitude is adjusted using the optical heterodyne detection setup. A DC voltage source additionally enables optical mode hybridization using stress-optic effects via the second actuator.

Pulsed pumping is implemented by driving an acoustic-optic modulator that is switched by a transistor-transistor logic signal generated by an arbitrary waveform generator. The pulse sequence is chosen such that the optical resonance does not drift significantly within each pulse, as indicated by a constant steady-state "pulse-on" transmission. Pulsed up-conversion is recorded utilizing the same heterodyne setup as continuous-wave up-conversion. During pulsed down-conversion experiments, the microwave output from the RF probes are routed to a lock-in demodulation setup for detection of weak pulses.

---

[1] H. Haus and W. Huang, Proceedings of the IEEE **79**, 1505 (1991).

[2] T. Blésin, H. Tian, S. A. Bhave, and T. J. Kippenberg, Phys. Rev. A **104**, 052601 (2021).

[3] C. M. Caves, Physical Review D **26**, 1817 (1982).

[4] A. Rueda, W. Hease, S. Barzanjeh, and J. M. Fink, npj Quantum Inf. **5**, 1 (2019).

[5] C. Zhong, Z. Wang, C. Zou, M. Zhang, X. Han, W. Fu, M. Xu, S. Shankar, M. H. Devoret, H. X. Tang, and L. Jiang, Phys. Rev. Lett. **124**, 010511 (2020).

[6] S. Krastanov, H. Raniwala, J. Holzgrafe, K. Jacobs, M. Lončar, M. J. Reagor, and D. R. Englund, Phys. Rev. Lett. **127**, 040503 (2021).

[7] L.-M. Duan, M. D. Lukin, J. I. Cirac, and P. Zoller, Nature **414**, 413 (2001).

[8] M. H. P. Pfeiffer, J. Liu, A. S. Raja, T. Morais, B. Ghadiani, and T. J. Kippenberg, Optica **5**, 884 (2018).

[9] J. Liu, G. Huang, R. N. Wang, J. He, A. S. Raja, T. Liu, N. J. Engelsen, and T. J. Kippenberg, Nat. Commun. **12**, 2236 (2021).

[10] H. Tian, J. Liu, B. Dong, J. C. Skehan, M. Zervas, T. J. Kippenberg, and S. A. Bhave, Nat. Commun. **11**, 3073 (2020).

[11] A. Siddharth, A. Anastasio, G. Lihachev, J. Zhang, Z. Qiu, S. Kenning, R. N. Wang, S. A. Bhave, J. Riemensberger, and T. J. Kippenberg, Hertz-linewidth and frequency-agile photonic integrated extended-DBR lasers (2023), arxiv:2306.03184 [physics].

[12] H. Tian, J. Liu, A. Siddharth, R. N. Wang, T. Blésin, J. He, T. J. Kippenberg, and S. A. Bhave, Nat. Photon. **15**, 828 (2021).

[13] M. Gao, Q.-F. Yang, Q.-X. Ji, H. Wang, L. Wu, B. Shen, J. Liu, G. Huang, L. Chang, W. Xie, S.-P. Yu, S. B. Papp, J. E. Bowers, T. J. Kippenberg, and K. J. Vahala, Nat. Commun. **13**, 3323 (2022).

[14] V. B. Braginsky, M. L. Gorodetsky, and V. S. Ilchenko, in *Laser Applications*, Vol. 2097 (SPIE, 1994) pp. 283–288.

[15] A. Schliesser, P. Del'Haye, N. Nooshi, K. J. Vahala, and T. J. Kippenberg, Phys. Rev. Lett. **97**, 243905 (2006).

[16] K. Leong and J. Mazierska, IEEE Trans. Microw. Theory Tech. **50**, 2115 (2002).

[17] J. Larson, P. Bradley, S. Wartenberg, and R. Ruby, in *2000 IEEE Ultrasonics Symposium. Proceedings. An International Symposium (Cat. No.00CH37121)*, Vol. 1 (2000) pp. 863–868 vol.1.

[18] T. Carmon, L. Yang, and K. J. Vahala, Optics Express **12**, 4742 (2004).
